# Clinical implementation of endolysins targeting gram-positive bacteria points toward a combination strategy with standard-of-care antibiotics: a selective review

**DOI:** 10.1186/s40001-025-03655-4

**Published:** 2026-01-09

**Authors:** Niels Vander Elst

**Affiliations:** Department of Neuroscience, Karolinska Institutet, Biomedicum 7D, Solnavägen 9 Solna, 17165 Stockholm, Sweden

**Keywords:** AMR, Antibiotic resistance, Antibiotic-resistant infection, Antibiotics, Beta-lactam, Bacteriophage-derived endolysin, Clinical, Endolysin, Glycopeptide, Gram-positive, Lipopeptide

## Abstract

Endolysins, which are peptidoglycan hydrolases derived from bacteriophages, are expected to innovate antimicrobial treatment. More specifically, several endolysins that target gram-positive bacteria are currently being evaluated in clinical trials, reflecting increasing interest in their therapeutic application. Research involving endolysins has expanded exponentially over the last 20 years, which has resulted in a substantial diversification. With most of the field having focused on endolysin discovery, biochemical characterization and applying protein engineering strategies, it remains unclear whether endolysins should eventually be implemented as stand-alone antimicrobials or alongside standard-of-care antibiotics, an ambiguity that is also reflected in the endolysins that are currently being evaluated in clinical trials. This selective review, inspired by a selection of preclinical studies in which endolysin monotherapy had inconsistent outcomes, concludes that endolysins hold their greatest therapeutic potential when used in combination with standard-of-care antibiotics, except in cases, where therapy is limited to a local or topical application only. In the latter, antibiotic supplementation can negatively impact the microbiome that is essential to maintain, for example, skin, ear, eye, nasopharyngeal, gut and vaginal homeostasis. By combining endolysins with antibiotics that preferably target the bacterial cell wall or membrane, such as β-lactams, as well as lipo- and glycopeptides, synergistic or additive effects can be exploited that substantially reduce minimal inhibitory concentrations, restoring antibiotic susceptibility in otherwise resistant bacteria. Overall, this selective review asserts that endolysins may be best implemented alongside standard-of-care antibiotics, as this may lead to more consistent and reliable clinical outcomes, particularly in systemic infections.

## Background

**Fig. 1:**
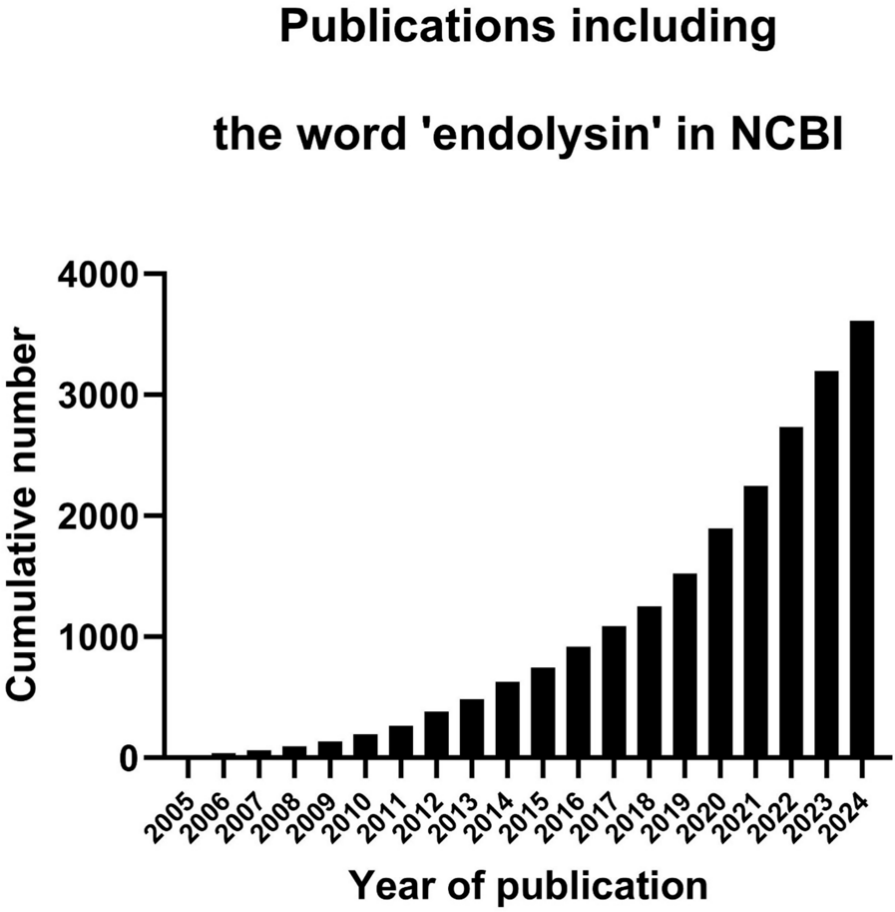
Cumulative number of publications in NCBI mentioning the term ‘endolysin’ from 2005 to 2024. Data retrieved from NCBI (query term: ‘endolysin’) up to 2024-12-31. The growth is exponential

Bacteriophage-encoded endolysins are enzymes that have been discovered already in the 1950 s [1], but it was only at the start of the new millennium that the first study was published which investigated their therapeutic potential as antimicrobials [2]. Endolysins are peptidoglycan hydrolases, produced by bacteriophages at the end of their lytic replication cycle, that aim the release of newly assembled virions causing ‘lysis from within’ [3]. In this ‘lysis from within’ scenario, the endolysin’s hydrolysing activity is tightly regulated through a secondary protein known as a holin, which form pores in the bacterial membrane and subsequently allow the endolysin to access and hydrolyse the peptidoglycan layer of the infected host [4]. However, endolysins retain their hydrolytic (lytic) activity when recombinantly produced in the laboratory and applied externally to gram-positive bacteria. This is referred to as the ‘lysis from without’ scenario [5]. In the latter, endolysins kill gram-positive bacteria by degrading the peptidoglycan layer, leading to the extrusion of intracellular contents due to high internal osmotic pressure, eventually resulting in death by osmotic lysis. Because endolysins have a different mechanism of action than antibiotics, they are particularly effective against antibiotic-resistant gram-positive bacteria, regardless of the resistance mechanisms that those bacteria have acquired [6]. This latter feature has led to an exponential growth of the literature on endolysins, particularly since 2005 (Fig. 1). While this rapid expansion has greatly diversified the field, it has also introduced inconsistencies in endolysin nomenclature [5, 7].

In-line with this rapid expansion, several endolysins have advanced from preclinical development into clinical trials, which includes XZ.700 (Micreos; phase I/IIa ongoing), SAL200 (iNtRON Biotechnology; phase II terminated early), CF-301 (ContraFect; phase III terminated early), LMN-201 (Lumen Bioscience; phase II ongoing), P128 (GangaGen, phase I/II completed) and HY-133 (HYpharm GmbH, phase I ongoing) [8–14]. In addition, SA.100 (Micreos) has already been marketed under the name Staphefekt, albeit registered as a cosmetic [15, 16]. Remarkably, XZ.700, P128 and HY-133 are being evaluated as stand-alone antimicrobials against *Staphylococcus aureus* for either local treatment of skin infections (atopic dermatitis; XZ.700) or nasal decolonization (P128 and HY-133), whereas SAL200, CF‑301, and LMN‑201 are being, or have been, assessed in combination with standard-of-care antibiotics for other indications, including (methicillin-resistant) *S. aureus* (MRSA) bacteraemia (SAL200 and CF‑301), MRSA-related right-sided endocarditis (CF‑301), and recurrent colitis caused by *Clostridioides difficile* (LMN‑201).

Overall, this diversified literature as well as these varying strategies for the clinical evaluation of endolysins, specifically in the absence or presence of standard-of-care antibiotics, have resulted in an ambiguity about their optimal pathway for advancing from the preclinical stage into clinical trials. This selective review examines current approaches and concludes, as supported by preclinical evidence, that the optimal strategy is to advance endolysins to a clinical setting in combination with standard-of-care antibiotics, particularly those targeting the bacterial cell envelope (i.e., β-lactams, lipopeptides, and glycopeptides). An important exception relates to applications, where preserving a beneficial microbiome is essential, such as treatments of the skin (e.g., atopic dermatitis), outer ear (e.g., otitis externa), eye (e.g., scleritis), nasopharynx (e.g., pharyngitis), gut (e.g., colitis), or vagina (e.g., vaginal dysbiosis). While preclinical studies have established proof of concept for endolysin monotherapy in systemic infections and beyond, this review seeks to identify and advocate for strategies to bridge the gap between preclinical research and clinical evaluation that could enhance the consistency and effectiveness of clinical outcomes of endolysin-based therapies even more.

## Methodology

This work is a selective review of literature retrieved from PubMed, Google Scholar, and ClinicalTrials.gov, covering approximately the last 25 years (January 2000–October 2025) using the search terms ‘endolysin’ and ‘phage-derived endolysin’. Articles were selected by the author based on relevance, with particular emphasis on studies investigating combinatory approaches of endolysins with antibiotics. The review focuses exclusively on Gram‑positive bacteria, particularly *Streptococcus* and *Staphylococcus* species, reflecting the author’s expertise. This selective approach may have introduced selection bias. To the best of the author’s knowledge, no endolysin targeting gram-negative bacteria has yet reached advanced human clinical trials.

## Review

### Current endolysins evaluated in clinical trials or on the market

As a starting point, valuable insights can be gained by examining endolysins that have already progressed into clinical development or reached the market. Understanding the therapeutic approaches employed, such as endolysin monotherapy versus combination strategies with standard-of-care antibiotics, and how these relate to specific clinical applications, is essential to formulate an optimal strategy for the future clinical implementation of other endolysin-based compounds. Moreover, as some trials have been prematurely terminated, identifying the factors that contributed to these early discontinuations is critical. Therefore, this selective review first provides an overview of endolysins that have undergone clinical evaluation or are already marketed, as summarized in Table 1, which are hereafter discussed in corresponding order. It should be noted that, in these clinical trials, the choice of antibiotic used in combination with endolysins is not specified, as therapy is typically individualized for each patient based on clinical evaluation (as mentioned in NCT03089697, NCT04160468, NCT05330182).

**Table 1:** Overview of endolysin-based products that are or have been evaluated in clinical trials, together with the developing company, the indication, combination strategy with antibiotics, clinical development stage with ClinicalTrials.gov identifier (if available), as well as trial status

Compound	Other names	Developer	Indication	Adjunct to antibiotic therapy	Stage (ClinicalTrials.gov identifier)	Trial status
XZ.700	N/A	Micreos	*S. aureus* dermatitis	No	Phase I/IIa (No identifier)	Ongoing
SAL200	SAL-1, Tonabacase, N-Rephasin	iNtRON Biotechnology	*S. aureus* bacteraemia	Yes	Phase II (NCT03089697)	Terminated Early
CF-301	PlySs2, Exebacase	ContraFect Corporation	*S. aureus* bacteraemia, Right-sided endocarditis	Yes	Phase III (NCT04160468)	Terminated early
LMN-201	LHD	Lumen Bioscience	Recurrent *C. difficile* colitis	Yes	Phase II (NCT05330182)	Ongoing
P128	StaphTAME, Staphtame	GangaGen	Nasal decolonization of *S. aureus*	No	Phase I/II (NCT01746654)	Completed
HY‑133	N/A	HYpharm GmbH	Nasal decolonization of *S. aureus*	No	Phase I (NCT06290557)	Ongoing
SA.100	Staphefekt	Micreos (Gladskin brand)	Cosmetic (acne vulgaris, eczema)	No	Market	Marketed

Antibiotic combinations are not further detailed, as the antibiotic choice in each combination is individualized for each patient based on clinical evaluation. P128 combines a virion-associated peptidoglycan hydrolase from Phage K with the lysostaphin SH3b cell-wall binding domain and is not a true endolysin, but mechanistically similar. SA.100 (Staphefekt) is marketed as a cosmetic and was not evaluated in clinical trials. N/A indicates not applicable

Micreos developed XZ.700, an engineered endolysin targeting *S. aureus* that combines the M23 peptidase domain from lysostaphin with the amidase and SH3b cell wall-binding domain from Ply2638 [15]. Furthermore, XZ.700 has demonstrated potent antibacterial activity in preclinical models of skin infection, specifically on reconstituted human skin and in murine models of skin infection. Currently, XZ.700 is in a Phase I/IIa clinical trial (no ClinicalTrials.gov identifier) for mild to moderate atopic dermatitis and is being evaluated as a topical monotherapy without standard-of-care antibiotics. Preclinical studies also suggest its potential for treating cutaneous T-cell lymphoma by reducing *S. aureus* colonization and malignant T cell activation [17]. Importantly, XZ.700 selectively targets *S. aureus* while sparing beneficial skin commensals, such as *Staphylococcus epidermidis*, helping preserve and restore microbial diversity and promoting wound healing, as demonstrated in porcine models [18]. Beyond skin infections, XZ.700 has shown efficacy in disrupting *S. aureus* biofilms on titanium surfaces without cytotoxic effects on human osteocyte-like cells, indicating it can potentially also be used as a coating for orthopaedic implants [19].

SAL200 (also known as SAL-1, Tonabacase, and N-Rephasin) is a wild-type endolysin developed by iNtRON Biotechnology for the treatment of systemic infections, particularly bacteraemia caused by *S*. *aureus*, including methicillin-resistant strains (MRSA). Preclinical studies have shown that SAL200 exhibits potent bacteriolytic activity against both methicillin-susceptible and -resistant *S. aureus* isolates and significantly reduced *S. aureus* loads in the blood and spleen of mice [9]. The greatest reductions were observed when SAL200 was used in combination with the standard-of-care antibiotics nafcillin (a β-lactam) and vancomycin. SAL200 completed a Phase I clinical trial (NCT01855048) in healthy volunteers, following dose optimization in monkeys, and demonstrated a favourable safety profile and pharmacokinetics after intravenous administration [20, 21]. However, anti-drug antibodies were detected in 10 out of 27 participants, with levels correlating to the administered endolysin dose. In a subsequent Phase II clinical trial (NCT03089697) conducted in South Korea, SAL200 was evaluated in patients with *S. aureus* bacteraemia as an adjunct to standard-of-care antibiotics. While the compound was well-tolerated, enrolment into the study was terminated by the sponsor prior to completion for strategic reasons.

CF-301 (a.k.a. PlySs2 or Exebacase) is one of the most extensively studied endolysins, demonstrating broad and potent lytic activity against a range of staphylococcal and streptococcal species in vivo [22–26]. Developed by ContraFect, CF-301 was positioned as a leading candidate for treating bacteraemia and right-heart endocarditis caused by MRSA, as well as chronic prosthetic joint infections [26, 27]. Despite promising results, ContraFect recently filed for bankruptcy, and the Phase III clinical trials (NCT04160468) for bacteraemia treatment were discontinued due to insufficient statistical power [12]. The company noted that this outcome may have been influenced by a disparity in baseline disease severity, with the CF-301 treatment group containing more patients with severe prognoses (APACHE II score > 15), potentially affecting mortality outcomes. It is important to note that CF-301 was administered exclusively as an adjunct to standard-of-care antibiotics. Beyond systemic infections, CF-301 has shown strong biofilm-disrupting capabilities on diverse surfaces, including polystyrene, glass, surgical mesh, catheters, and within human synovial fluid [28, 29]. Therefore, CF-301 was also evaluated in patients with chronic knee prosthetic joint infections, where the infected implant was replaced through a so-called LysinDAIR procedure, in which CF-301 was infused into the knee joint alongside standard-of-care antibiotics at the end of the surgery, resulting in clinical improvement [11, 27]. Remarkably, CF-301’s antibacterial potency was significantly enhanced, by 32- to over 100-fold, in human blood compared to standard laboratory media across multiple testing methods (MIC, time-kill assays, and synergy assays) [30]. This enhanced activity is partially attributed to CF-301’s synergistic interactions with human blood components, particularly serum lysozyme and albumin. The synergy with lysozyme is thought to result from CF-301’s initial peptidoglycan disruption, which sensitizes bacteria to lysozyme’s enzymatic action, while both CF-301 and *S. aureus* exhibit high affinity for albumin, facilitating their co-localization and enhancing CF-301’s bactericidal effect in the bloodstream.

LMN-201 is a therapeutic cocktail developed by Lumen Bioscience aimed at treating recurrent colitis caused by *C. difficile*. The cocktail includes an engineered endolysin that combines the catalytic domain of the bacteriophage phiC2 endolysin with the functional domain of a human defensin protein, which also exhibits antimicrobial activity [10]. In total, the cocktail contains four proteins: the engineered endolysin and three additional proteins designed to bind and eliminate the major toxins of *C. difficile*, which are the primary virulence factors responsible for disease pathology [31]. Preclinical studies demonstrated potent bactericidal activity of the engineered endolysin against diverse *C. difficile* strains, as well as effective mitigation of symptoms and reduced mortality in a mouse model [10]. The lysin-containing compound has been optimized for enhanced stability and activity in the gastrointestinal environment, including resistance to proteolytic degradation and acidic pH, although specific details of these modifications have not been publicly disclosed yet. Currently, LMN-201 is being evaluated in Phase II clinical trials (NCT05330182) to prevent recurrent *C. difficile* infections, where it is administered alongside standard-of-care antibiotics [8].

P128 (a.k.a. StaphTAME or Staphtame) is a peptidoglycan hydrolase developed by GangaGen, designed to decolonize the nares of *S. aureus*, including methicillin- and vancomycin-resistant strains. It is a chimeric protein combining a virion-associated peptidoglycan hydrolase from phage K with the lysostaphin SH3b cell-wall binding domain (CBD), and is, therefore, not a true endolysin, but mechanistically similar [14]. Preclinical studies demonstrated potent bacteriolytic activity against *S. aureus*, including MRSA, as well as reductions in minimum biofilm inhibitory concentration (MBIC) when combined with conventional antibiotics, such as vancomycin, gentamicin, ciprofloxacin, linezolid and daptomycin [32]. Moreover, P128 improved survival in rats that were systemically infected with *S. aureus* when applied as a monotherapy, and reduced CFU counts to the limit of detection in a rat nasal colonization model (in 4 out of 9 animals) [14, 33]. P128 progressed to Phase I/II clinical trials (NCT01746654) as a nasal spray, without antibiotic supplementation, to evaluate safety and efficacy in healthy volunteers, and in chronic kidney disease patients identified as *S. aureus* nasal carriers. These Phase I/II clinical trials have been completed, but to the author’s knowledge, their results have not yet been published, and no Phase III trial has been announced.

HY-133, developed by HYpharm GmbH, is an engineered endolysin that combines the cysteine- and histidine-dependent aminopeptidase/hydrolase (CHAP) catalytic domain from the endolysin of phage K (LysK) with the lysostaphin SH3b CBD incorporating a shortened linker peptide which enhances stability against protease inactivation [34]. This optimized construct exhibits potent bacteriolytic activity against *S. aureus*, including MRSA, as well as difficult-to-treat small-colony variants [35–37]. Moreover, HY-133 has been shown to eradicate *S. aureus* from vascular graft surfaces while displaying no cytotoxicity toward primary human endothelial or vascular cells [38, 39]. Pharmaceutical formulation efforts have focused on developing nasal hydrogel/ointment and sprayable preparations for topical (intranasal) application without antibiotic supplementation [13]. HY-133 has entered Phase I clinical trials (NCT06290557) for nasal decolonization of *S. aureus*, which are currently ongoing.

SA.100 (a.k.a. Staphefekt) is an engineered endolysin developed by Micreos that is commercially available in Gladskin products designed to target *S. aureus*-associated skin conditions, including acne vulgaris and eczema. Notably, SA.100 and XZ.700 share the same domain composition, that is, the M23 peptidase domain from lysostaphin combined with the amidase and SH3b cell wall-binding domain from Ply2638, although XZ.700 is a linker-improved variant of SA.100 [15] Case studies involving three patients have suggested that regular use of Staphefekt-containing products can reduce *S. aureus* colonization on the skin and potentially alleviate symptoms of *S. aureus*-driven skin inflammation (dermatosis), potentially without disrupting beneficial skin commensals [16]. However, given its registration as a cosmetic rather than a medicinal product, SA.100 is currently positioned primarily for microbiome modulation and skincare, rather than for the actual treatment of active *S. aureus* skin infections.

### Challenges of endolysins in clinical settings and preclinical efforts addressing those

**Fig. 2:**
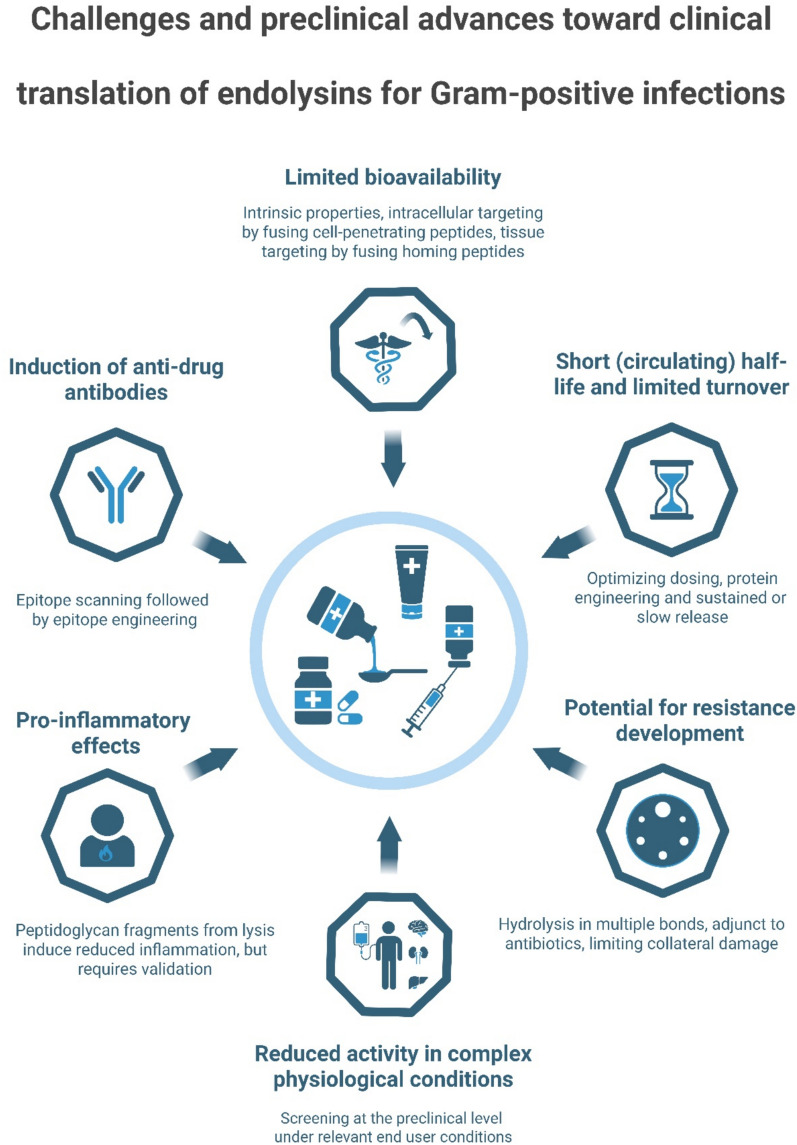
Schematic overview of the challenges that may limit the therapeutic translation of endolysins, including: (i) short (circulating) half-life, (ii) reduced activity in complex physiological conditions, (iii) pro-inflammatory effects due to bacterial antigen release, (iv) limited bioavailability, (v) induction of anti-drug antibodies, and (vi) potential for resistance development. Solutions validated at the preclinical level include optimized dosing and formulation strategies, protein engineering approaches (e.g., domain or peptide fusions, dimerization, glycosylation, and epitope modification), targeted delivery via cell-penetrating or homing peptides, and screening under end user conditions

Although this review focuses specifically on endolysins targeting gram-positive bacteria, it is worth mentioning that several other peptidoglycan hydrolases, most notably lysozyme and lysostaphin (a bacteriocin), share a similar modular architecture and catalytic mechanism [40, 41]. These enzymes were investigated as antimicrobials already decades earlier but never progressed to advanced clinical development or market approval. Lysostaphin, for example, was tested in a human patient for *S. aureus* infection in 1974, although at the time not within a formal clinical trial [42]. The (pre)clinical development of these enzymes predates that of endolysins and offers valuable insights into challenges that are also relevant to the clinical translation of endolysins. The limited clinical translation of these enzymes at the time (in the 1960 s and 1970 s) may be partly explained by the lower perceived urgency of antimicrobial resistance, as well as the limited economic incentives for protein-based therapeutics, which had to compete with conventional antibiotics that were cheaper and easier to produce at that time [43]. Although the clinical development of lysozyme and lysostaphin was limited by these practical and economic factors, several mechanistic and pharmacological barriers identified for these enzymes can be recognized as general challenges that also constrain the therapeutic translation of endolysins, being: (i) short (circulating) half-life, (ii) reduced activity in complex physiological conditions, (iii) pro-inflammatory effects due to bacterial antigen release, (iv) limited bioavailability, (v) induction of anti-drug antibodies, and (vi) the potential for resistance development [44]. For endolysins, however, many of these challenges have now been addressed at the preclinical level, resulting in concrete solutions that are discussed hereafter alongside each of these 6 aforementioned challenges (Fig. 2):

(i) Endolysins are enzymes that target peptidoglycan as a substrate, thus converting it into a hydrolysed product. According to basic biocatalysis principles, enzymes are limited by the amount of substrate they can convert, known as the turnover number (k_cat_). Thus, unlike antibiotics, endolysins may not remain functional for as long as they persist inside the patient. In addition, being proteins, endolysins may be susceptible to degradation by serine proteases present at the site of infection and mainly produced by the innate immune system (neutrophils) to help fight the invading bacterial pathogen [45, 46]. Furthermore, when administered systemically, endolysins are primarily eliminated through renal clearance [19], which can further reduce their bioavailability at the target site. Overall, this results in a short (circulating) half-life of endolysins, which has been confirmed for the well-studied pneumococcal Cpl-1 endolysin. Cpl-1 was reported to have a half-life of only 16 and 20 min in cerebrospinal fluid and plasma, respectively, following intracisternal and intraperitoneal injection in rats and mice [47, 48]. Notably, the authors reported the recurrence of pneumococci in CSF after 4 h following a single intracisternal administration of Cpl-1 and reasoned this to be caused by the short half-life specifically. To address this challenge, the half-life of endolysins, particularly the circulating half-life after systemic administration, has been extended through the optimization of dosing regimens in vivo, as well as through various engineering strategies. For example, the staphylococcal endolysin SAL200 (a.k.a. SAL-1) demonstrated a maximum half-life of 38 min in healthy human volunteers after optimizing the intravenous dosing strategy in monkeys [20, 21]. Engineering strategies to increase the half-life in circulation have been successfully applied to the staphylococcal endolysin LysK by fusing an albumin binding domain (utilizing so-called masking strategies) [49], or by increasing the hydrodynamic volume through glycosylation or dimerization, shown for lysostaphin and the pneumococcal Cpl-1 endolysin, respectively [50, 51]. Another interesting strategy involves the addition of an N-terminal cysteine residue that enables chemical conjugation with C16 palmitic acid, resulting in reversible serum albumin binding without impacting the antibacterial activity, but increasing the circulating half-life by approximately fourfold [52]. Other solutions to extending the half-life of endolysins may lie in formulation strategies designed to achieve sustained or slow release [53].
(ii) Endolysins are nearly always initially characterized under laboratory conditions in buffers that favour their lytic activity [54]. As a result, it is common that endolysins, which initially have been characterized with activity under such laboratory conditions, fail to provide therapeutic efficacy in proof-of-concept or clinical studies. One such example comes from veterinary medicine in which an application with endolysins has been aimed for bovine mastitis, an infection of the cow’s udder, which requires the evaluated endolysins to be active in raw milk [3]. Multiple groups have done extensive screening assays to characterize endolysins with activity against streptococci or staphylococci in pasteurized milk, but when the selected candidates were tested in raw milk, which is substantially different from pasteurized milk, activity was frequently reduced, thus contrasting the initial results in pasteurized milk [55, 56]. Furthermore, it was also reported that endolysins have reduced activity against biofilms grown in milk compared to those grown under regular laboratory conditions [57]. Taken together, these studies have clearly demonstrated that screening for endolysins, usually happening for large panels after high-throughput engineering [58, 59], must happen under the end user conditions (e.g., matrix, pH, ionic strength, and presence of proteases) [5, 54, 55, 60]. On the other hand, some endolysins, such as the pneumococcal endolysin Cpl-1, have been successfully characterized with potent activity in human blood and in human cerebrospinal fluid without initially screening for variants that display activity in such conditions [61]. Furthermore, synergy with serum lysozyme and co-localization on albumin have been reported in human serum for the staphylococcal endolysin CF-301 (a.k.a. PlySs2), indicating that antimicrobial activity determined under laboratory conditions may sometimes also be underestimated [30].
(iii) An inevitable consequence of the mode of action of endolysins is the release of intracellular bacterial components and cell wall fragments, which may trigger pro-inflammatory responses by acting as pathogen-associated molecular patterns (PAMPs) [62]. Similar concerns have been raised for bactericidal antibiotics, which, in contrast to bacteriostatic antibiotics, promote the release of intra-bacterial components and can have devastating consequences, particularly in infections of the central nervous system [61, 63]. Augmented pro-inflammatory responses following endolysin treatment compared to antibiotic treatment have been reported for the well-known pneumococcal endolysin Cpl-1 in the context of *S. pneumoniae* endocarditis [64]. In a rat model of pneumococcal endocarditis, the authors compared high-dose, continuous Cpl-1 treatment to vancomycin and observed increased serum levels of IL-1α, IL-1β, IL-6, IFN-γ, and TNF-α. This enhanced pro-inflammatory response was suggested to result from an increased release of cell wall fragments following Cpl-1 treatment [64]. In addition, other authors reported a trend, although not statistically significant, toward increased Iba-1 staining (indicating microglial activation) in the brains of mice that were treated with Cpl-1 endolysin applied either as a monotherapy and in combination with penicillin, compared to other groups that did not receive the endolysin [61]. Although the release of intracellular bacterial components and cell wall fragments remains an important challenge, it has contrastingly been shown for lysostaphin that rapid extracellular lysis of *S. aureus* releases peptidoglycan fragments that are insufficient to fully activate TLR2-dependent inflammatory responses, as effective cytokine induction requires specific three-dimensional motifs or intracellular processing within phagosomes, which is suggested to be circumvented by lysostaphin-mediated lysis [65]. Thus, endolysin-mediated peptidoglycan hydrolysis may elicit a comparatively reduced pro-inflammatory response, but this requires further experimental validation for endolysins specifically.
(iv) A key challenge in using endolysins systemically is their reduced bioavailability in peripheral tissues and limited ability to cross tissue barriers, which limits access particularly to intracellularly protected bacteria [55]. However, several endolysins have demonstrated intrinsic capabilities to traverse eukaryotic cell membranes and lyse intracellular pathogens. For example, PlyC exhibits intracellular activity against *Streptococcus pyogenes* by interacting with phosphatidylcholine, normally located on the inner leaflet of the plasma membrane but transiently exposed during a process of cell membrane recycling, internalizing PlyC and co-localizing with intracellular bacteria [66]. Furthermore, the pneumococcal endolysin Cpl-1 has been shown to cross the blood–brain barrier (BBB), hypothesized to happen via co-transport of choline by the platelet-activating factor receptor, providing protection against meningoencephalitis caused by invasive pneumococcal disease [61]. As a concrete solution to target intracellular bacteria, engineering strategies have been developed to enhance the cellular uptake and tissue penetration of endolysins. These include fusion to cell-penetrating peptides, such as the HIV-1 TAT peptide [55, 67, 68], or incorporation of tissue-targeting moieties (so-called homing peptides), for instance, to direct endolysins toward osteoblasts in bone infections, thereby increasing local concentrations and facilitating eradication of intracellular bacteria [69]. Notably, some endolysins already possess intrinsic cell-penetrating properties; for example, the SH3b domain of CF-301 (a.k.a. PlySs2) has been shown to mediate membrane translocation through four amino acid residues that function as an intrinsic cell-penetrating peptide [70].
(v) An inevitable consequence of using biologicals as therapeutic drugs is the induction of anti-drug antibodies (ADAs), which are known to be produced against endolysins in humans, as was reported following intravenous injection of SAL200 (a.k.a. SAL-1) [21]. The induction of ADAs, specifically IgE and IgG, may lead to adverse reactions toward endolysin treatment, the neutralization of the endolysin’s antibacterial activity, and a reduced (circulating) half-life. However, studies in mice have shown that anti-endolysin IgE is not always induced, and IgG may not always be neutralizing, as in some cases IgG antibodies have been reported to bind outside the catalytic pocket [71, 72]. More specifically, in mouse studies evaluating the streptococcal endolysin PlyC and the pneumococcal endolysins Cpl-1 and Pal, IgE levels remained at baseline, while a gradual increase in IgG levels was expected. In humans, SAL200 (a.k.a. SAL-1) demonstrated the occurrence of ADAs in 10 out of 27 participants in the study, with levels proportional to the endolysin dose received [21]. Whereas ADA formation following endolysin treatment appears inevitable, a promising solution seems the targeted modification of endolysins as recently demonstrated for the pneumococcal Pal endolysin [73]. Through epitope scanning and *in silico* engineering, a variant was created that had a significantly enhanced antibacterial activity, but more importantly, prevented cross-neutralization by ADAs induced by wild-type Pal endolysin. This demonstrates a promising solution to mitigate neutralizing IgG ADAs after initial endolysin treatment, which could help to reduce immunogenicity and drug inactivation.
(vi) Another challenge that has been raised is the potential development of resistance toward endolysins. This reasoning follows resistance observed for lysozyme, which breaks the glycan backbone in staphylococcal peptidoglycan. It is known that *Staphylococcus* can O-acetylate muramic acid in the peptidoglycan resulting in resistance toward lysozyme [74]. Contrastingly, staphylococci (e.g., *S. aureus* and *S. epidermidis*) have been challenged and passaged with increasing concentrations of endolysin (e.g., LysRODI), but resistance development was not observed even after 10 passages [75]. Several reasons have been proposed why it is unlikely that gram-positive bacteria can acquire resistance against endolysins. The first one relates to the endolysin’s short half-life, meaning that a bacterial population is only temporarily exposed to the selective pressure of an endolysin [5]. The second reason is that endolysins usually, but not always, contain multiple catalytic domains (e.g., amidase, muramidase, and peptidase) and thus hydrolyse the peptidoglycan in multiple bonds, in contrast to lysozyme and lysostaphin that rely on hydrolysing a single major bond type, being β−1,4 glycosidic linkages and the pentaglycine cross-bridges, respectively [76]. Another reason might be that endolysins limit collateral damage by staying bound to the peptidoglycan of the bacterial cell that was lysed. Experimental proof of the latter comes from certain endolysins (e.g., LysK) which have been truncated to their catalytic domain only, thus deleting the cell wall binding domain, and surprisingly having an increased lytic activity by increasing the enzymatic turnover number (k_cat_) [77].

### Endolysins may have inconsistent therapeutic outcomes if applied as stand-alone antimicrobials, particularly in systemic infections

Extensive preclinical evidence supports the use of endolysins as monotherapies [78], but it must also be acknowledged that these outcomes have not been consistently observed across all studies, particularly concerning systemic infections [44]. In fact, several endolysins evaluated in various settings have demonstrated limited therapeutic efficacy when used alone. This inconsistency in endolysin monotherapy potency suggests a need to consider strategies that promote a more reliable and robust therapeutic outcome, thus increasing the likelihood of clinical success. One such promising strategy that comes into focus is the combined clinical implementation of endolysins alongside standard-of-care antibiotics, particularly in systemic infections, which is the key message of this selective review. The following examples illustrate preclinical cases, where endolysin monotherapy showed limited or inconsistent efficacy:

(i) The pneumococcal endolysin Cpl-1, one of the best-characterized endolysins to date, was evaluated as a monotherapy in a rat model of pneumococcal meningitis and found that a single dose of Cpl-1 did not save nor protect rats from pneumococcal meningitis [47]. After pneumococcal meningitis was established, 600 µg Cpl-1 was injected into the cranium which reduced pneumococci below the limit of detection (i.e., 10^4^ CFU/mL), but in less than 2 h, pneumococci started to re-appear, which the authors reasoned to be caused by either bacterial regrowth or new pneumococci invading the brain from the blood. Importantly, the half-life of endolysin Cpl-1 in cerebrospinal fluid was found to be only 16 min, a factor previously discussed as a key challenge impacting the sustained efficacy of endolysins in systemic applications. In addition, another study assessed the neuroprotective effect of the Cpl-1 endolysin in human neuronal-like cells infected with penicillin-resistant pneumococcal clinical isolates [61]. Despite serial administration of Cpl-1 endolysin up to three times, the rapid exponential growth of pneumococci outpaced the antibacterial activity of the endolysin, resulting in continued bacterial proliferation and subsequent neuronal death, demonstrating that Cpl-1 monotherapy also here had inconsistent outcomes. In contrast, combining the Cpl-1 endolysin with penicillin restored the penicillin susceptibility of the pneumococci after only one dose, resulting in full neuroprotection, an effect that was thereafter also validated in a mouse model of pneumococcal meningitis [61].
(ii) Endolysin NC5, an engineered enzyme incorporating the TAT peptide from HIV-1 to facilitate intracellular killing of pathogens within bovine mammary epithelial cells that was selected from over 80,000 variants, demonstrated variable efficacy in raw milk [55]. NC5 demonstrated potent activity against bovine mastitis-causing streptococci in pasteurized milk, but when NC5 was compared to cloxacillin treatment in raw milk samples obtained from cows with *S. uberis* mastitis, the results showed that in only one case (1 out of 4), its activity was not inhibited by the raw milk matrix. Thus, no significant improvement compared to classical cloxacillin treatment was observed (3 out of 4). In contrast, when endolysin NC5 was evaluated together with cloxacillin, this combination consistently and significantly killed *S. uberis* faster in all raw milk samples (4 out of 4), a strategy that was subsequently also validated in a mouse model of streptococcal mastitis [79]. As a reminder, this study was also previously discussed to illustrate the challenge endolysins face in maintaining activity in complex physiological conditions, thus demonstrating the necessity to screen panels of endolysins under relevant end-user conditions [5].
(iii) Like the previous two examples, the staphylococcal endolysin SAL200 (a.k.a. SAL-1), currently in clinical trials for *S. aureus* bacteraemia alongside standard-of-care antibiotics, has also demonstrated inconsistent efficacy as a monotherapy [9]. Whereas SAL200 exhibited promising bactericidal activity *in vitro*, regrowth was frequently observed within the first few hours following endolysin monotherapy. However, when combined with cell-envelope-targeting antibiotics, such as nafcillin (a β-lactam) or vancomycin (a glycopeptide), SAL200 showed synergistic or additive effects, which suppressed *S. aureus* growth more effectively than the endolysin monotherapy. In a subsequent mouse model of systemic *S. aureus* infection, SAL200 monotherapy reduced bacterial loads in the blood and spleen, but the combination therapies consistently achieved even greater reductions.

### Endolysins can restore the antibiotic effect in resistant bacteria by potentiating antibiotic treatment through additive or synergistic effects

Since the discovery of penicillin, antibiotics have become indispensable for controlling bacterial infections and they remain so today, despite rising resistance levels. Antibiotics used to treat bacterial infections can be broadly categorized into three main classes based on their (intra)cellular targets: (i) inhibitors of cell envelope synthesis, (ii) inhibitors of protein synthesis, and (iii) inhibitors of DNA synthesis. Below, selected examples of endolysins in combination with antibiotics are presented according to this broad classification, summarized in Table 2, to explore their potential in providing even greater consistency in therapeutic outcomes compared to endolysin monotherapy. Among these, combinations with antibiotics targeting the bacterial cell envelope appear particularly promising, as synergistic effects are frequently reported and have extensively been validated in preclinical *in vivo* models:

(i) The selected examples discussed first are combination strategies involving cell-envelope-targeting antibiotics, which are mainly (a) β-lactams (e.g., penicillin), (b) lipopeptides (e.g., daptomycin) and (c) glycopeptides (e.g., vancomycin). (a) Starting with β-lactams, synergy with endolysins has been frequently reported given that both can act on the bacterial peptidoglycan directly. For example, already in 2005 it was reported that the pneumococcal Cpl-1 endolysin reduced the MIC value of penicillin [6]. This synergistic action between penicillin and Cpl-1 was later confirmed by another group, who importantly also noted that this synergistic action reduces MIC values below the clinical breakpoint for penicillin resistance [61]. More specifically, penicillin and Cpl-1 endolysin at doses that were not protective individually in mice, could protect synergistically both on a systemic level (blood, spleen) as well as in the brain [61]. Identical observations were also obtained for: oxacillin with the engineered endolysin ClyS and also with CF-301 (a.k.a. PlySs2), as well as nafcillin with SAL200 (a.k.a. SAL-1), all in the context of methicillin-resistant *S. aureus* (MRSA) bacteraemia [9, 80, 81]; cloxacillin with the engineered endolysin NC5 in the context of streptococcal mastitis [55, 79]; amoxicillin and cefotaxime with endolysin Cpl-711 in the context of pneumococcal bacteraemia [82], and more [5]. (b) To proceed with the lipopeptides, which target the bacterial membrane, synergy has similarly been reported between daptomycin and the pneumococcal Cpl-1 endolysin [83]. Corresponding to the potentiating effect as observed for β-lactam antibiotics, a synergistic combination of daptomycin with Cpl-1 endolysin saved 80% of mice with pneumococcal bacteraemia. In comparison, no mice survived with Cpl-1 monotherapy, and only 35% survived with daptomycin alone. Another observation has been made for the staphylococcal endolysin CF-301 (a.k.a. PlySs2), in which a synergistic combination with daptomycin significantly improved survival to 73%, whereas treatment with CF-301 resulted in 13% survival and daptomycin alone achieved 23% [81]. (c) Vancomycin is a well-known and widely used glycopeptide, considered critical for health care by the WHO given that it is frequently used as last resort when other antibiotics fail [84]. Vancomycin inhibits bacterial cell wall synthesis by binding to the D-Ala–D-Ala peptide motif of the peptidoglycan precursor, thereby disrupting cell wall formation. The synergistic potential of vancomycin in combination with endolysins has been extensively studied, specifically in the context of *S. aureus* infections [e.g., for LysP108, CF-301 (a.k.a. PlySs2) and SAL200 (a.k.a. SAL-1)] [9, 81, 85]. LysP108 exhibited synergy with vancomycin against a vancomycin-resistant MRSA isolate. This synergy was demonstrated both in vitro using a checkerboard assay and in a mouse abscess model, where the combination treatment, in comparison with antibiotic and endolysin monotherapy, resulted in the fastest abscess resolution [85]. Similarly, synergy has been observed between vancomycin and the well-characterized staphylococcal endolysin CF-301 (a.k.a. PlySs2) at sub-MIC vancomycin concentrations. Using checkerboard assays, synergy was detected for 18 out of 26 MRSA strains, and for 25 out of 29 methicillin-susceptible *S. aureus* (MSSA) strains [81]. This finding was validated in a mouse model of *S. aureus* bacteraemia, where MRSA-infected mice received treatment 2 h post-inoculation with CF-301 (a.k.a. PlySs2), vancomycin, or their combination. At 72 h, survival rates were 3% for both monotherapies, whereas combination therapy significantly improved survival of the mice to 67%. Taken together, these studies demonstrate that combining cell-envelope-targeting antibiotics with endolysins offers a more consistent and effective antimicrobial strategy compared to endolysin monotherapy, restoring the antibiotic effect by exploiting synergistic or additive effects and lowering minimal inhibitory concentrations, potentially even below clinical resistance breakpoints.
(ii) Antibiotics used for infections caused by gram-positive bacteria that inhibit the bacterial protein synthesis involve among other macrolides (e.g., erythromycin) and tetracyclines (e.g., doxycycline) by targeting the 50S and 30S subunits of the bacterial ribosomes, respectively. One study evaluated the interaction between erythromycin and the pneumococcal Cpl-1 endolysin in the case of macrolide-resistant *S. pneumoniae* and reported that additive effects were present [61]. More specifically, MIC values were frequently lowered below clinical breakpoints for macrolide resistance, thus restoring erythromycin susceptibility in resistant pneumococcal clinical isolates. However, the number of studies that have investigated the interaction between endolysins and antibiotics that target the bacterial protein synthesis are limited and can be proposed as a focus point for future research.
(iii) Antibiotics that inhibit bacterial DNA synthesis, such as the (fluoro) quinolones, are more commonly used to treat infections caused by gram-negative rather than gram-positive bacteria and thus fall beyond the scope of this selective review that wants to focus on gram-positive bacteria only. However, certain (fluoro) quinolones, such as levofloxacin, exhibit coverage against gram-positive bacteria, particularly against *Streptococcus pneumoniae*, and are, therefore, sometimes used to treat pneumococcal pneumonia or invasive disease in the case of resistance to other antibiotic classes [84]. From this perspective, the combination of levofloxacin with the pneumococcal Cpl-711 engineered endolysin (comprising the catalytic domain of Cpl-7 and the cell wall-binding domain of Cpl-1) has been investigated in both levofloxacin-sensitive and -resistant strains. The results demonstrated either additive or synergistic effects, depending on the bacterial isolate [82]. However, the most pronounced synergistic activity was observed with other antibiotics that target the bacterial cell envelope, namely, amoxicillin and cefotaxime (both β-lactams). This once again demonstrates that an optimal combinatory approach may lie in combining endolysins with antibiotics that target the bacterial cell envelope.

**Table 2:** Preclinical evaluations of endolysins combined with antibiotics, organized by antibiotic class, antibiotic, target pathogen, endolysin, observed effect (synergistic or additive) with FIC (fractional inhibitory concentration) range, if reported, and study reference

Antibiotic class	Antibiotic	Pathogen	Endolysin	Observed effect (FIC range, if available)	Study
Beta-lactam	Penicillin	*S. pneumoniae*	Cpl-1	Synergistic or additive (0.25–1.67)	Djurkovic S. et al., 2005 [6]
Beta-lactam	Penicillin	*S. pneumoniae*	Cpl-1	Synergistic (0.25–0.50)	Vander Elst N. et al., 2025 [61]
Beta-lactam	Oxacillin	*S. aureus*	ClyS	Synergistic (< 0.50)	Daniel A. et al., 2010 [80]
Beta-lactam	Oxacillin	*S. aureus*	CF-301 (a.k.a. PlySs2)	Synergistic or additive	Schuch R. et al., 2014 [81]
Beta-lactam	Nafcillin	*S. aureus*	SAL200 (a.k.a.SAL-1)	Synergistic or additive (0.38–0.53)	Kim N.H. et al., 2018 [9]
Beta-lactam	Cloxacillin	*S. uberis*	NC5	Synergistic or additive	Vander Elst N. et al., 2023 [55]
Beta-lactam	Amoxicillin	*S. pneumoniae*	Cpl-711	Synergistic or additive (0.43–0.91)	Letrado P. et al., 2018 [82]
Beta-lactam	Cefotaxime	*S. pneumoniae*	Cpl-711	Synergistic or additive (0.50–0.87)	Letrado P. et al., 2018 [82]
Lipopeptide	Daptomycin	*S. pneumoniae*	Cpl-1	Synergistic or additive	Vouillamoz J. et al., 2013 [83]
Lipopeptide	Daptomycin	*S. aureus*	CF-301 (a.k.a. PlySs2)	Synergistic or additive	Schuch R. et al., 2014 [81]
Glycopeptide	Vancomycin	*S. aureus*	LysP109	Synergistic	Lu Y. et al., 2021 [85]
Glycopeptide	Vancomycin	*S. aureus*	CF-301 (a.k.a. PlySs2)	Synergistic or additive	Schuch R. et al., 2014 [81]
Glycopeptide	Vancomycin	*S. aureus*	SAL200 (a.k.a.SAL-1)	Synergistic or additive (0.38–0.56)	Kim N.H. et al., 2018 [9]
Macrolide	Erythromycin	*S. pneumoniae*	Cpl-1	Additive (1.032–1.125)	Vander Elst N. et al., 2025 [61]
(Fluoro)quinolones	Levofloxacin	*S. pneumoniae*	Cpl-711	Synergistic or additive (0.40–0.75)	Letrado P. et al., 2018 [82]

The FIC index quantifies the interaction between two antimicrobials: synergistic (FIC < 0.5), additive (0.5–4.0), or antagonistic (> 4.0). Reported effects should be interpreted cautiously, as assay conditions, bacterial strains, and protocols varied across studies. No antagonistic effects were reported

### Endolysins must be used as stand-alone antimicrobials in localized infections to protect the beneficial microbiome

In localized infections, such as those affecting the skin, external ear, eye, nasopharynx, gut or vagina, a combination approach with antibiotics may not be the most appropriate strategy for endolysins [2, 15, 53, 86–88]. In such settings, co-administration of antibiotics could even be counterproductive, given the critical role of the local microbiota in tissue repair and homeostasis. Compared to antibiotics, endolysins offer a distinct advantage through their high specificity, allowing for the selective elimination of pathogenic bacteria while preserving the surrounding beneficial microbiota [88]. From this perspective, they have been proposed as ‘microbiome modulators’ that can reshape microbial communities without the collateral damage typically associated with antibiotic treatment. This selective activity should be maximally exploited in cases, where infection is localized, and the integrity of the microbiota is essential to host health. Several studies have investigated this potential and are discussed below to illustrate how microbiome-sparing strategies with endolysins may be applied in different localized infections.

A first example concerns Staphefekt SA.100, the first endolysin to become commercially available, marketed in Gladskin products by Micreos [16, 44]. Case studies in humans have shown significant improvement in chronic skin conditions associated with *S. aureus* dermatosis following treatment with Staphefekt SA.100 without antibiotic supplementation [16]. Participants applied a Staphefekt SA.100-containing cream twice daily, without antibiotic supplementation, and all reported clear clinical improvements that had not been achieved prior with antibiotic treatments. Micreos also reported that another of their staphylococcal endolysin, being XZ.700 that currently is in Phase IIa clinical trials, selectively inhibited endogenous *S. aureus* on the skin of pigs, restoring microbial diversity and promoting multiple aspects of wound repair [18].

A second example is PlyC, an endolysin that targets group A streptococci (GAS) also referred to as *Streptococcus pyogenes*. GAS can reside in the nasopharynx and are typically associated with streptococcal pharyngitis. In vitro studies demonstrated that PlyC lysed a diverse panel of 10 GAS strains to the limit of detection within 5 min, with no viable colonies detected after treatment [2]. In a subsequent murine colonization model, oral administration of PlyC as a monotherapy effectively prevented colonization and eliminated *S. pyogenes* from the upper respiratory tract after only one single dose. However, more importantly, when PlyC was tested against streptococci that are part of the nasopharyngeal microbiome, such as *S. crista*, *S. intermedius*, *S. gordonii*, *S. mitis*, *S. mutans*, *S. oralis*, *S. parasanguis* and *S. salivarius*, PlyC showed either none or very minimal lytic activity toward these strains.

A third example is the engineered endolysin PM-477, designed to target *Gardnerella vaginalis*, a gram-positive-like opportunistic pathogen that often overgrows in vaginal dysbiosis. PM-477 demonstrated high efficacy in eliminating *G. vaginalis* while leaving beneficial lactobacilli and other vaginal bacteria unaffected [89]. Its effectiveness was assessed as a monotherapy in vaginal samples from fifteen patients with either first-time or recurrent bacterial vaginosis. In thirteen cases, PM-477 successfully eradicated *G. vaginalis* while preserving the rest of the vaginal microbiome.

## Conclusions

Endolysins are currently under preclinical development, in various stages of clinical trials or already available on the market [8]. The latter comprises SA.100 only, and it is important to emphasize that this product is registered as a cosmetic, which differs substantially from registration as a medicinal product [15, 16]. Endolysins that are being or have been evaluated in clinical trials include XZ.700 (Micreos; phase I/IIa ongoing), SAL200 (iNtRON Biotechnology; phase II terminated early), CF-301 (ContraFect; phase III terminated early), LMN-201 (Lumen Bioscience; phase II ongoing), P128 (GangaGen, phase I/II completed) and HY-133 (HYpharm GmbH, phase I ongoing) [8–14]. This selective review aims to formulate an optimal strategy to advance endolysin-based therapeutics from preclinical evaluation into clinical trials, primarily by relying on preclinical studies and existing clinical approaches employed for the aforementioned endolysins. While extensive preclinical evidence supports endolysins as effective monotherapies [78], several studies have also reported inconsistent outcomes in preclinical models [9, 47, 55, 61]. These inconsistencies highlight the need for a more reliable strategy to achieve even greater consistent therapeutic results, an important consideration as additional endolysins are expected to progress into clinical trials. This review also identifies key challenges associated with the clinical application of endolysins, which are the short (circulating) half-life, reduced activity in complex physiological conditions, pro-inflammatory effects due to bacterial antigen release, limited bioavailability, induction of anti-drug antibodies, and potential for resistance development. Although many of these challenges are being or have been partially addressed at the preclinical level, they may still have contributed to early clinical trial terminations, alongside other factors such as variability in baseline disease severity leading to insufficient statistical power (CF-301) [12], and strategic decisions (SAL200). From that perspective, this selective review wants to advocate for combination strategies in which endolysins are paired with standard-of-care antibiotics, as this strategy enables some of these aforementioned challenges to be mitigated. Indeed, combining endolysins with antibiotics can help circumvent bacterial regrowth once the endolysin’s (circulating) half-life has expired, even so in the case of antibiotic resistance [61]. This is because combination therapy can restore the antibiotic effect by lowering the minimum inhibitory concentration, even below clinical resistance breakpoints, a result of synergistic or additive effects. In addition, combination therapy makes it more difficult for bacteria to develop resistance simultaneously to two different classes of antimicrobials. However, some challenges still require further investigation or validation, particularly the inflammatory response caused by bacterial antigen release, which may be especially problematic in infections involving the central nervous system, where inflammation can have severe consequences [90]. As for the other remaining challenges, the development of ADAs as well as reduced bioavailability may potentially be addressed through protein engineering strategies [55, 69, 73], whereas reduced activity in complex physiological conditions could be overcome by screening panels of (engineered) endolysins as close as possible to the end user conditions [5, 54, 55].

Combination strategies proved most promising with antibiotics that target the bacterial cell envelope, such as β-lactams (e.g., penicillin), lipopeptides (e.g., daptomycin) and glycopeptides (e.g., vancomycin) [61, 81, 83]. Synergistic effects are more likely to arise here, because both agents act on the bacterial cell envelope directly, amplifying their overall bactericidal activity. In contrast, it may be more challenging to achieve the same extent of synergy in combinations with antibiotics that target the bacterial protein or DNA synthesis, given that these antibiotics do not directly share the same molecular target as endolysins [61]. However, additive effects can be expected, because endolysins weaken the bacterial cell wall, thus facilitating the entry of intracellularly acting antibiotics to their targets. As an important side note, the extent of synergy may also vary as it most likely also depends on the antibiotic resistance mechanisms present, the antibiotic susceptibility, and the bacterial sensitivity toward the endolysin itself [6]. Most studies to date have assessed synergy or additive effects between endolysins and a limited range of antibiotics, but a thorough comparative analysis to identify key factors driving synergy remains lacking and can be proposed as a focus point for future research.

One key exception to the proposed combination approach concerns infections in which a beneficial microbiome is present. In such cases, endolysins should be used as stand-alone antimicrobials to maximize their narrow-spectrum bactericidal effects, selectively targeting pathogenic bacteria while preserving the beneficial microbiota [88]. In addition, they can also serve as precise ‘microbiome modulators’, reshaping microbial communities without the broad collateral damage that is typically associated with conventional antibiotics. This latter reasoning likely explains why XZ.700, P128 and HY-133 are being evaluated in clinical trials without antibiotics, as the intended application is *S. aureus* skin infections (e.g., atopic dermatitis) for the former (XZ.700) and nasal *S. aureus* decolonization for the two latter (P128 and HY-133), thus aiming to preserve the protective and repairing effect of the microbiome [18]. This is in contrast with SAL200 and CF-301 that are intended for systemic *S. aureus* infections (SAL200 and CF-301) and chronic prosthetic joint infections (CF-301), which are being evaluated in combination strategies [9, 26, 81]. Another possible explanation to proceed with a combinatory strategy may also lie in the regulatory pathway, as it is often more feasible to register new biologicals as adjuncts to existing therapies rather than as standalone replacements. However, a deviation from this microbiome-sparing reasoning is the clinical trial ongoing with LMN-201 (NCT05330182), currently being evaluated in combination with standard-of-care antibiotics for the prevention of recurrent colitis caused by *C. difficile*, thus using a combination strategy in the presence of the gut microbiome. This combinatory approach may also be motivated by the inherently challenging environment of the gut, which can reduce endolysin activity under complex physiological conditions. The ongoing clinical trial (NCT05330182) explicitly states that the primary objective of LMN-201 is to prevent recurrence of disease following *C. difficile* infection, thus meaning the aim is not to replace antibiotic treatment during the acute phase of the disease. In addition, the choice of a combination strategy for LMN-201 may also similarly reflect regulatory considerations.

**Fig. 3:**
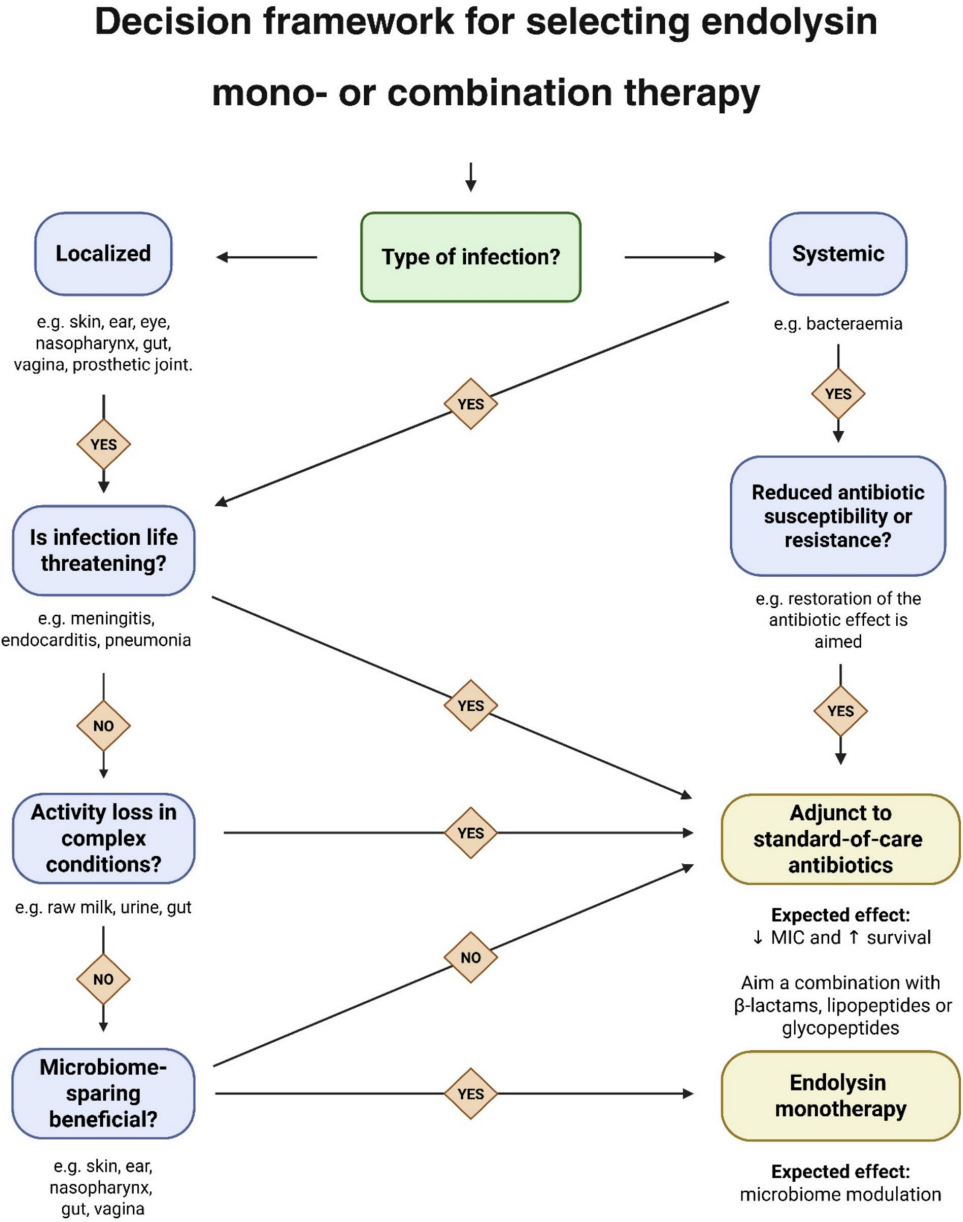
Decision framework for selecting endolysin mono- or combination therapy toward clinical implementation. The framework guides the choice between endolysin monotherapy and combination therapy based on infection type, severity, and treatment goals. Systemic or antibiotic-resistant infections favor combination therapy with standard-of-care antibiotics, while localized, non-life-threatening infections, where microbiome preservation is desirable, are better suited for endolysin monotherapy

Collectively, these findings emphasize that the therapeutic use of endolysins must be carefully tailored to the infection type, localization, and clinical context. As a guideline, Fig. 3 illustrates a conceptual decision framework for selecting endolysin mono- or combination therapy for the clinical implementation of endolysin-based products, informed by the many factors that were identified in this review.

In conclusion, this selective review advocates for a dual-path strategy in the clinical development of endolysin-based antimicrobials: leveraging endolysins as monotherapies in microbiome-sensitive infections, while prioritizing combination approaches, particularly with cell-envelope-targeting antibiotics, for systemic infections. This approach may help maximize their current clinical potential by providing more consistent therapeutic outcomes than endolysin monotherapy while addressing the urgent need for novel and effective antimicrobial strategies.

## Data Availability

The datasets used and/or analysed during the current study are available from the corresponding author on reasonable request.

## Declarations

### Artificial intelligence

Text editing support was provided using AI-assisted tools (ChatGPT, OpenAI) to improve clarity and language precision.
